# Assessing the impact of water use in conventional and organic carrot production in Poland

**DOI:** 10.1038/s41598-022-07531-7

**Published:** 2022-03-03

**Authors:** Zbigniew Kowalczyk, Maciej Kuboń

**Affiliations:** Faculty of Production and Power Engineering, University of Agriculture in Cracow, ul. Balicka 116B, 30-149 Kraków, Poland

**Keywords:** Environmental impact, Agroecology

## Abstract

As global water resources are decreasing and the demand for it is constantly increasing, the problem of proper water management is becoming more pressing. Poland is one of the largest producers of vegetables in Europe, including carrots, with significant exports. However its freshwater resources are relatively small. The paper presents the results of research on the water footprint (WF) life cycle assessment (LCA) in conventional and organic carrot production. The methodology of calculating WF was used in accordance with PN-EN ISO 14046. It was found, e.g., that WF for organic production of carrot (WF = 1.9 m^3^ ha^−1^) is over five times lower, as compared to conventional production (WF = 10.4 m^3^ ha^−1^). In the case of conventional production, the fertilization process (67.0–67.7%) has the greatest impact on the shaping of WF in the individual impact categories, i.e. Human Health, Ecosystem Quality and resources. In organic production, the WF-shaping factor is carrot harvesting (41.9–43.1%). The research can be used to develop pro-ecological carrot production technologies, as well as to shape sustainable development plans in agricultural areas. It can also be used to outline policy directions regarding foreign trade in water-consuming agricultural products.

## Introduction

As one of the most important natural resources, water is essential for maintaining the integrity of ecosystems and for the development of human society and economy^1–5^. One of the significant threats to the global economy and to the human population is the constantly diminishing freshwater resources and the progressive water deficit in ever larger areas of the globe^6–9^. In parallel with resource depletion, human demand for water has increased almost eightfold in the last 100 years^10^.

Until recently, freshwater resources, its availability, use, and management were dealt with mainly at local and national levels^11^. For many years, the focus has been on the impact of climate change on freshwater resources, and the societal impact on the water management issues was ignored^12^. The recognition of the problem of accelerating changes in water availability, as well as the impact of globalization on water management, has prompted many researchers to raise the issue of freshwater resources and their consumption in a global context^11^.

When assessing water resources, the quality of available water is also essential, in addition to its quantity, as studied by many researchers^13–17^. The worldwide increase in environmental awareness among agricultural producers and consumers has sparked interest in WF of agricultural production. WF is a direct and indirect water consumption index introduced into the water management science to demonstrate the importance of consumption patterns and the global dimension of proper water management^11,18^. WF considerations have gained momentum as the ISO 14046 standards were published^19^, which encouraged certification activities in the food production system^20^. The importance of water consumption in the context of life cycle assessment (LCA) was mentioned many years ago^21–26^. The very concept of WF is an indicator of direct and indirect water use^18^. The WF LCA methodology considers the total amount of water used by a production system, from cradle (raw material extraction) to grave (waste management)^27^.

Water management of individual countries is very often based on a purely national perspective, striving to match domestic water demand with national water resources and generally disregarding the global dimension of water management. Many countries trade in water-intensive goods, but not all governments are interested in saving water by importing water-intensive products or using the relative abundance of water to produce water-intensive goods for export^11^. Understanding and calculating WF of agricultural products is essential for the development of a well-thought-out national agricultural, economic and foreign trade policy. A rational international trade in water-intensive products can help reduce water scarcity or can reduce water consumption in water-scarce regions^28,29^. One of the largest consumers of water in the world is agriculture^30–33^. In fact, water abundance is one of the main factors increasing land productivity, agricultural productivity and, consequently food security^34^. Moreover, as 70% of the freshwater consumed by humans is used in agriculture, it is therefore essential to better understand the complex relationship between water and agriculture^35^.

Poland is one of the largest agricultural producers in Europe, especially in regard to the production of vegetables, including carrots. Annually, 700–800,000 tons of carrots are produced, of which more than 30,000 tons are exported^36^. Therefore, the problem of the carrot WF in terms of its production and exports seems important, especially since Poland is one of the countries with relatively small freshwater resources, compared to other European countries, and approx. 2.5 times lower amount of water per capita than the European average^37^.

## Purpose and scope

Carrot production technologies vary depending on the production region, soil quality, and potential quality of the harvested crops (conventional and organic production), hence they are characterized by different consumption of production materials and the use of machines in the production process^38^. Therefore, it can be assumed that different production technologies have a different WF. This study aimed to analyze WF of conventional and organic carrot production in southern Poland in terms of LCA. The results were analyzed to indicate which stages of individual carrot production technologies have the greatest water consumption, and thus the environmental impact. The research covered 20 plantations, 10 of which are conventional and the other 10 organic, with WF analysis for the production area and for the harvested crops. According to Jolliet et al.^39^, WF analysis can be used to support the sustainable development goals, hence the results can be used to monitor progress in introducing sustainable production. In addition, WF is regarded as a serious assessment tool for the effects of the consumption of water-related goods and services^40^, and the obtained study results can serve this purpose by e.g., indicating which particularly water-intensive means of production used in carrot production should be produced in countries with high water availability, and imported thence.

## Materials and methods

### Subject of the research

The farms with carrot plantations included in the research are located in southern Poland, where high-quality soils are abundant. All farms have a long carrot production tradition and are equipped with specialized field work machinery. Carrot plantations on the same soil class and located in the same region were selected for the study to enable comparative analysis. The varieties of carrots used (Kalina, Kometa, and Anka) are the most common in Poland, and their yields ranged from 35 to 49 t ha^−1^ (40 t ha^−1^ on average) for organic production and 43–65 t ha^−1^ (54 t ha^−1^ on average) in conventional production. The average area of plantations is 0.94 ha for organic and 1.19 ha for conventional plantations. Both conventional and organic crops were not irrigated. The average transport distance is 0.9 km for organic plantations, and 1.2 km for conventional. The research covered 10 organic and 10 conventional plantations.

### System boundaries

**Figure 1:**
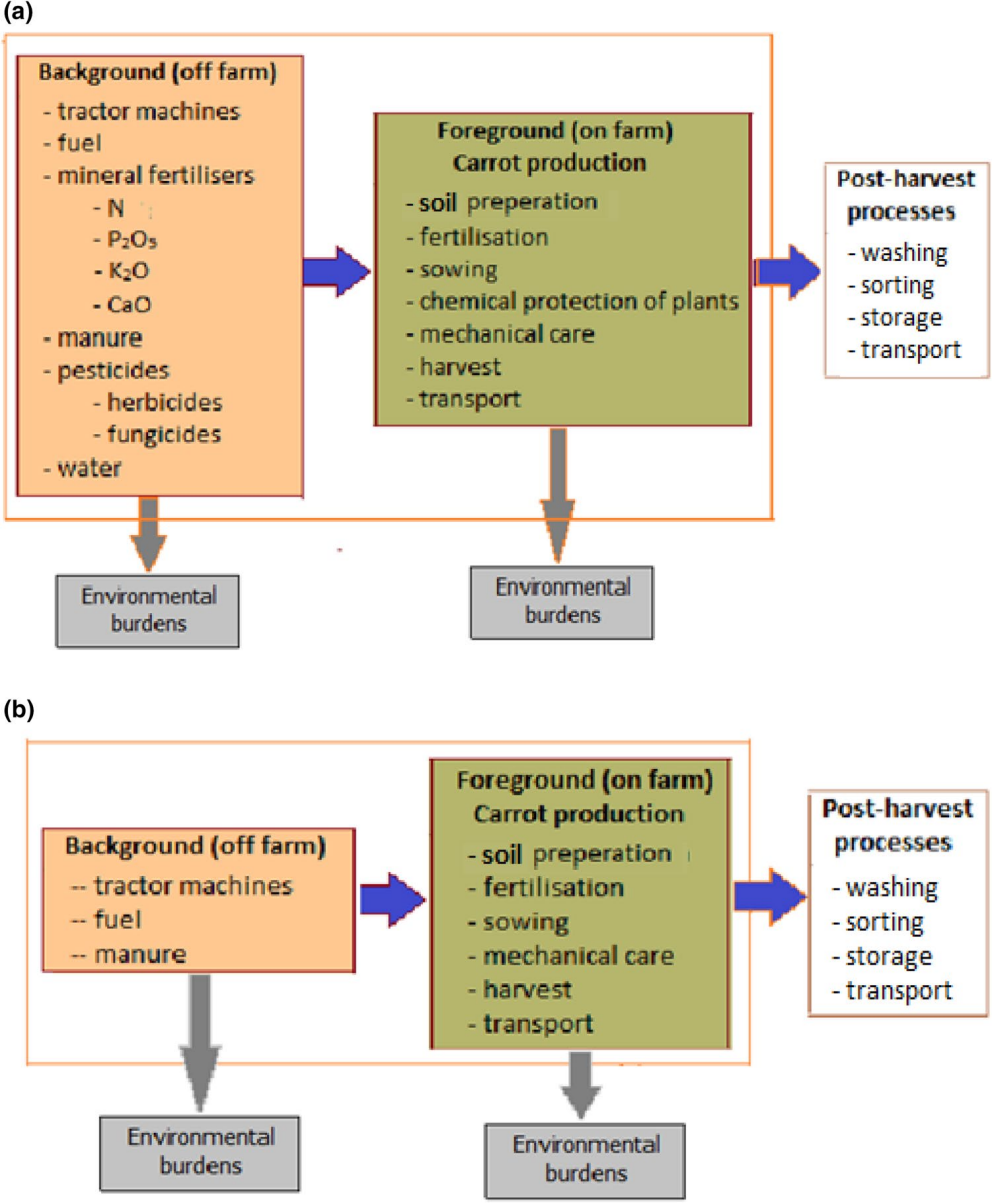
System boundaries: (**a**) conventional production of carrot, (**b**) organic production of carrot.

In the Life Cycle Assessment (LCA) methodology, a system boundary is defined to establish the test subject and what could possibly have been omitted. One of the most frequently used approaches in LCA research is the so-called “cradle-to-gate” method, i.e. taking into account all processes, from the extraction of raw materials from the ground (cradle), to transport, refining, processing and production, until the product is ready to leave the factory gate (in this case farm) to be transported to the consumer. Figure 1 shows the system boundaries for organic and conventional farming. In both cases, the cradle-to-gate approach was used, omitting the processes related to transporting the means of production to the farm and the post-harvest preparation of carrots for sale, and the sale process itself. The processes related to soil preparation, mineral and organic fertilization, sowing seeds, mechanical and chemical protection of plants, harvesting, and transport from the field to storage facilities on the farm were analyzed in detail.

### Water footprint calculation methodology

When analyzing water consumption in quantitative and environmental aspects, the applied LCA approach was used to assess the total consumption of resources in relation to environmental damage, i.e., water depletion^41^. LCA comprises four phases: goal definition and scoping, inventory analysis, impact assessment, and interpretation. Quantitative impact indicators are presented in the middle phase of the impact assessment^42^. As mentioned above, in the LCA methodology, studying the impact of water consumption in production processes takes into account the total amount of water used by the production system, from water required to source raw and production materials, to water required to manufacture and operate machinery involved in the production process, to water used directly in the production process, etc. In the LCA-based methodology, most existing methods quantify water scarcity by taking into account the ratio of water use to its availability and express it as the scarcity, or stress index^43^. This study evaluated the effects of midpoint and endpoint water intake; the midpoint water intake study used a detailed WF calculating methodology developed by Pfister et al.^44^.

The input data for the calculation of WF include withdrawal or consumption of water required by the product or process. They are adjusted using a factor defined as the water stress index (WSI)^44^.

$$\mathrm{WSI}= \frac{1}{1+ {e}^{-6.4 \cdot WTA} \cdot (\frac{1}{0.01}-1)}$$

WSI is estimated based on the withdrawal water to availability ratio (WTA) and modeled using a logistic function (S-curve) to fit the resulting index values between 0.01 and 1 m^3^ deprived/m^3^ consumed. The curve is adapted using OECD water stress thresholds that moderate and severe water stress as 20% and 40% of withdrawals, respectively. Water withdrawal and availability data were obtained from the WaterGap model. The index is applied to the volume of drinking water used. The environmental impact of endpoint water use was calculated according to the methodology by Pfister et al.^45^.

In endpoint analysis, the impact of water use is generally related to specific endpoints in a given conservation area: Human Health, Ecosystems Quality or Resources^43^. The impact of water use on human health is determined by modeling the causal chain of water scarcity (lack of irrigation water) leading to malnutrition, and is expressed in DALYs. Ecosystem quality is determined by modeling the causal chain of freshwater consumption impact on terrestrial ecosystem quality, and is assessed by species disappearing per year (species * year). The impact of water consumption in the Resources category, on the other hand, is determined by modeling the causal chain of freshwater consumption impact with respect to water depletion, and the unit is excess cost ($ surplus) of extracting an additional cubic meter of water^46^. Detailed computation was made using the SimaPro software, ver. 8.1.0.60, commonly used in LCA analyzes. The functional unit to which the obtained results were related was the carrot production area (1 ha). The tests, calculations, and analysis of the results were carried out in accordance with the recommendations of ISO 14046^19^, ISO 14040^47^ and ISO 14044^48^.

### Life Cycle Inventory (LCI)

Life Cycle Inventory (LCI) is a stage of the LCA methodology and involves creating an inventory of input and output data for all process units included in the assessment^23^: the flow of raw materials, materials, water, energy and emissions to soil, water and air. The input data used to calculate WF comes from detailed records kept during the study at 20 farms. During the research, individual carrot production technologies were analyzed in detail, with particular emphasis on the type of technological treatments and machines used, as well as their working time. The inputs (fertilizers, pesticides, water, fuel, etc.) and energy in individual carrot production technologies, as well as data selection, were taken with due diligence, according to ISO 14044^48^. Due to methodological difficulties in calculating the environmental impact of carrot seeds (not listed in the SimaPro software), the seed material was omitted. Following the calculation of the area of individual plantations and the volume of carrot harvest were calculated, the final results were compared against the adopted production area unit (1 ha).

In respective technologies, two types of farm tractors were usually used for field works. For light work, such as chemical spraying, weeding, or transport, tractors with approx. 26 kW were used, and for heavier work, mainly related to soil cultivation—tractors with approx. 56 kW. The technology of soil cultivation was usually quite similar: plowing was carried out in autumn with tractor plows. In the case of organic production, cattle or pig manure was applied before plowing. In the spring, other production treatments were applied: harrowing, cultivating and preparation of drills for sowing. Mineral fertilization was applied only in conventional crops, with the use of tractor-mounted centrifugal fertilizer spreaders. In the year of production, liming was applied only in one organic and one conventional plantation. Sowing was carried out with the use of tractor-mounted precision seeders. None of the researched plantations were irrigated as the rainfall was sufficient. In the case of organic crops, mechanical plant care was applied, i.e., weeding with tractor hoes. In organic plantations, weeding was largely also done by hand.

Chemical protection of the plants consisted in applying pesticides (mainly herbicides and fungicides) using tractor sprayers. The carrot was harvested in one or two stages. In single-stage harvesting, combine harvesters were used to dig up the carrots, remove the aboveground parts, clean and collect the roots. In the two-stage technology, mowers were used to first cut the carrot leaves, and then the harvesters dug up and cleaned the carrot roots. The carrot was transported in a similar manner, i.e., on agricultural tractors and trailers. The only difference was the greater amount of transport works, which in turn resulted in much higher carrot yield in conventional production. After transporting to the farm, the carrots were either sold or briefly stored. Post-harvest activities (sorting, washing, packing and shipping) basically did not differ regardless of the type of crop and were not included in the analysis. The data in Table 1 shows the consumption of means of production and the amount of transport works in both carrot production technologies in question, i.e., organic and conventional.

**Table 1:** Comparison of the consumption of selected means of production in conventional and organic carrot production per cropping area (ha).

Specification	Conventional cultivation	Organic cultivation
**Pesticides**		
Fungicides (kg)	2.95	0
Herbicides (kg)	2.55	0
**Mineral fertilizers**		
N (kg)	61	0
K_2_O (kg)	30	0
P_2_O_5_ (kg)	74	0
CaO (kg)	54	285
Manure (kg)	0	3778
Water (kg)	1173	0
Diesel (kg)	113	102
Transport (h)	8.5	7.6

## Results and discussion

**Figure 2:**
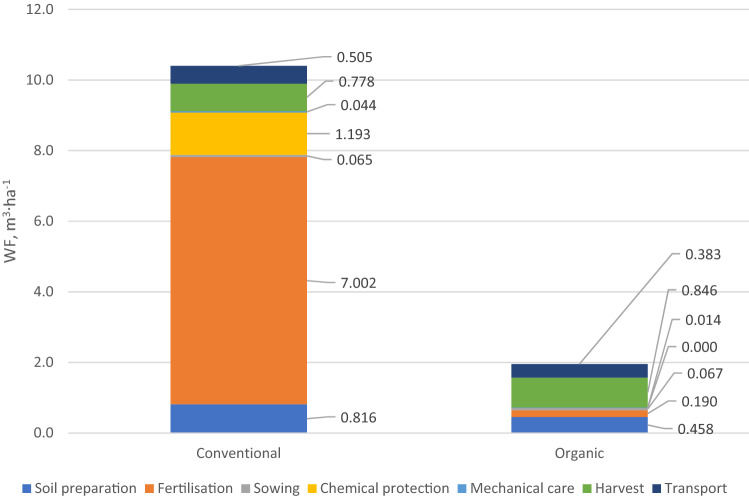
Water footprint in conventional and organic carrot production (m^3^ ha^−1^).

The LCA approach includes the potential effects of depriving humans and ecosystems of water resources, as well as the specific potential effects of pollutants affecting water and thus the environment^49^. Water stress is commonly defined as the ratio of total freshwater consumption to the level of its hydrological availability. ISO 14046 presents a new concept, i.e., WF, which is associated with the LCA approach. The standard's “water scarcity footprint” refers to the potential impacts associated with the quantitative aspect of water use^50^. Figure 2 shows the WF per cultivation area of conventional and organic carrot production. In general, there are significant differences in the total value of the WF in question. For conventional carrot production technology, it is 10.25 m^3^ ha^−1^, while for organic technology, it is only 1.96 m^3^ ha^−1^. In the case of conventional production, treatments using significant amounts of chemicals have the greatest impact on the WF, i.e., fertilization (mainly mineral) (WF = 6.85 m^3^ ha^−1^), and chemical plant protection (WF = 1.19 m^3^ ha^−1^). The analysis of WF in organic farming showed that its highest value (WF = 0.84 m^3^ ha^−1^) concerns the harvesting of carrots, while soil preparation ranks second (WF = 0.45 m^3^ ha^−1^). A slightly lower WF of 0.38 m^3^ ha^−1^ was recorded in the case of transporting the harvested carrots to the farm buildings. It can therefore be concluded that in organic farming, it is (diesel) fuel consumption that has the greatest impact on WF level.

In terms of production volume, in conventional technology, the WF is 0.196 m^3^ t^−1^. On the other hand, in organic technology the value of WF is approx. four times lower and amounts to 0.049 m^3^ t^−1^ of harvested carrots. For comparison, the WF in tomato production is 160 m^3^ per 1 tonne of produce^51^. Such a high value results mainly from irrigation of the plants.

**Figure 3:**
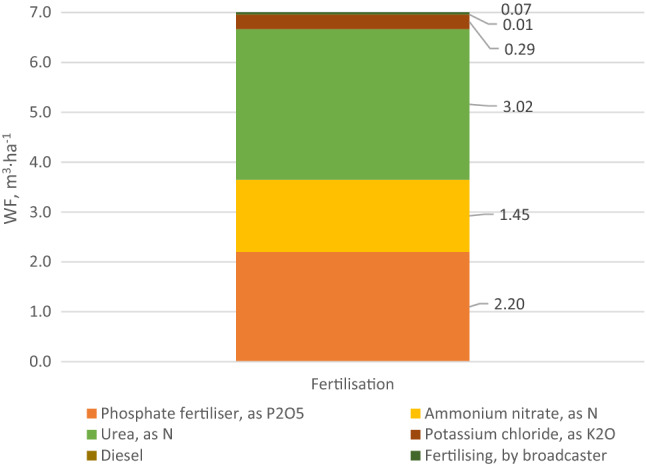
Water footprint related to carrot fertilization in conventional production (m^3^ ha^−1^).

In order to explain in detail the impact of individual agricultural treatments on the water deficit in carrot production, a detailed WF analysis was carried out for the treatments that demonstrated the highest values. In the case of conventional technology, it was fertilization (Fig. 3). Upon analyzing Fig. 3, it can be observed that the use of urea, and hence nitrogen, has the greatest impact on WF with regard to fertilization. Most nitrogen mineral fertilizers have a negative impact on the environment, causing ozone depletion in the stratosphere, groundwater pollution, global warming, and water eutrophication^52,53^. The largest water footprint associated with the use of mineral fertilizers in conventional cultivation is mainly due to a very energy-intensive fertilizer production process. Depending on the type of fertilizer and the technology used, the production process involves machines and equipment for cleaning, grinding, drying, sieving, extruding, granulating, packing, pumping, evaporation (crystallization) and transport. The vast majority of these treatments are powered by electricity. In contrast, conventional power production, regardless of the technology and fuel used (nuclear, natural gas, or coal), is characterized by very high water consumption. Mineral fertilizers are also a material whose consumed mass is relatively high compared to other production materials (seeds, pesticides, and diesel fuel). These two factors mentioned above have a decisive impact on the largest water footprint associated with the use of mineral fertilizers in conventional carrot cultivation. Processes requiring the use of machinery, i.e., fertilizer spreaders (1%) and the consumption of diesel fuel (0.1%) have the lowest impact on the level of fertilization-induced WF. Such a low impact of diesel fuel results mainly from its relatively low consumption during fertilization, most often using very efficient centrifugal spreaders. For comparison, WF related only to the use of carrot irrigation water is 20 m^3^ t^−1^ of harvested crops^54^.

**Figure 4:**
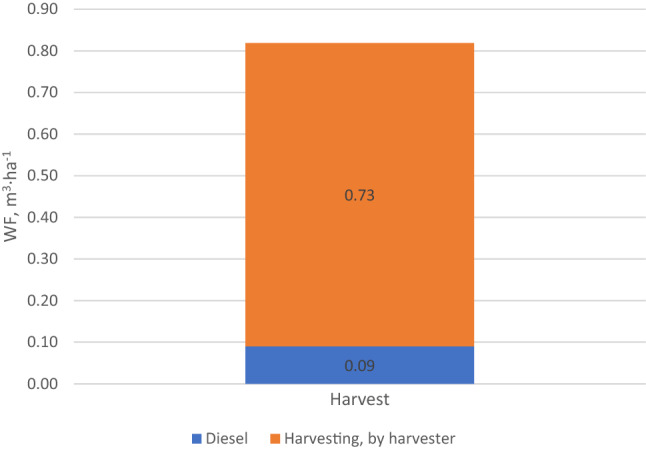
Water footprint related to carrot harvest in organic production (m^3^ ha^−1^).

In the case of organic technology, WF of harvesting was analyzed in detail (Fig. 4). Carrots were excavated with harvesters, which cut the aboveground parts, cleaned the roots and collected them in a hopper. Sometimes the excavation was preceded by mowing the carrot leaves with mowers. Carrot harvesters are machines that require farm tractors with high-power combustion engines, and the harvesting procedure itself is very time-consuming, hence such a large impact of fuel consumption on WF in carrot harvesting. Despite the above, the share of diesel consumption in the total value of WF related to carrot harvesting is only 11%. However, when comparing the WF related to fuel consumed during harvesting and during fertilization, it can be noticed that in the case of harvesting, WF is approx. 15 times higher.

**Figure 5:**
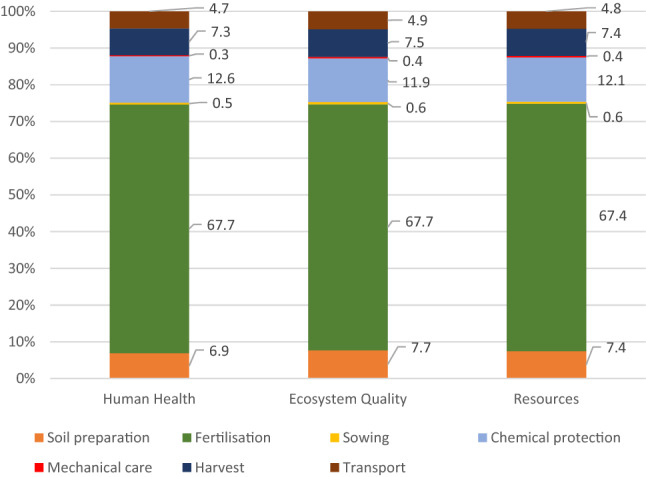
The structure of WF in individual impact categories in conventional carrot production.

In LCA, the potential effects of water pollution have traditionally been addressed in impact categories such as (eco) toxicity, acidification, and eutrophication^42,43^. In the WF analysis, the impact of water consumption is generally related to specific goals within a given conservation area, such as: Human Health, Ecosystems Quality and Resources^43^. The impact of water consumption on human health is expressed in DALY and is obtained by modeling the cause-effect chain of water scarcity (lack of irrigation water) leading to malnutrition. Ecosystem quality is assessed by modeling the cause-effect chain of freshwater consumption with the quality of the terrestrial ecosystem, based on the number of species disappearing each year (species * year). On the other hand, the impact of water consumption in the resources category is assessed by modeling the cause-effect chain of freshwater consumption in relation to the depletion of water resources, along with the cost ($) of extracting an additional cubic meter of water^46^. The data in Table 2 shows WF in conventional carrot production related to the three impact categories, and Fig. 5 shows its structure. The total impact of individual processes in the Human Health category is 1.15E−05 DALY, in the Ecosystem Quality category—1.53E−07 species * year, and in the Resources category—2.97 $ surplus. For comparison, WF in the above-mentioned impact areas per 1 ha of tomatoes is, respectively: Human Health—5.00E−03 DALY, Ecosystem Quality—2.50E−05 species * year^55^. When analyzing Fig. 5, it can be observed that in all impact categories, fertilization has the greatest environmental impact, the share of which in individual categories is at approx. 67.0–67.7%. Chemical plant protection ranks second, the impact of which in the three categories ranges from 11.9 to 12.6%. In addition to the treatments related to fertilizers and chemicals, treatments associated with high consumption of diesel fuel, i.e., soil preparation and harvest, have a significant impact on the value of individual categories in carrot production. This confirms the results of many studies, i.e. that the extraction, production and, above all, the use of diesel fuel bring significant damage to the environment^56,57^.

**Table 2:** Environmental impact related to the use of water in conventional carrot production per area unit (ha).

Specification	Total	Soil preparat.	Fertilization	Sowing	Chemical protection	Mechan. care	Harvest	Transport
Human health (DALY)	1.15E−05	7.98E−07	7.81E−06	5.39E−08	1.46E−06	3.89E−08	8.38E−07	5.40E−07
Ecosystem quality (species * year)	1.53E−07	1.18E−08	1.03E−07	9.51E−10	1.82E−08	6.00E−10	1.15E−08	7.47E−09
Resources ($ surplus)	2.97E+00	2.21E−01	2.00E+00	1.67E−02	3.59E−01	1.09E−02	2.19E−01	1.41E−01

Bearing in mind that carrot yield range in conventional cultivation is 43–65 t ha^−1^, the total impact of individual processes per 100 tons of harvested carrots is as follows: Human Health: 2.17E−05 DALY, Ecosystem Quality: 2.88E−07 species * year and Resources: 5.57 $ surplus.

Upon comparing the obtained results with the research presented in the literature and conducted with a similar methodology, it can be concluded that for the production of 100 tons of tomatoes, the total environmental footprint for the above-mentioned impact areas, including factors other than water, is respectively: Human Health: 2.7E−01 DALY, Ecosystems Quality: 1.45E−03 Species * year, Resources: 1.05E + 06 $^58^. On the other hand, the WF for green beans, per 100 tons of harvest was reported as follows: Human Health: from 2.00E−2 to 1.08E−1 DALY, Ecosystem Quality: from1.10E−3 to 1.80E−3 species * year, Resources: from 1.90E+2 to 1.40E+3 $ surplus^59^.

**Figure 6:**
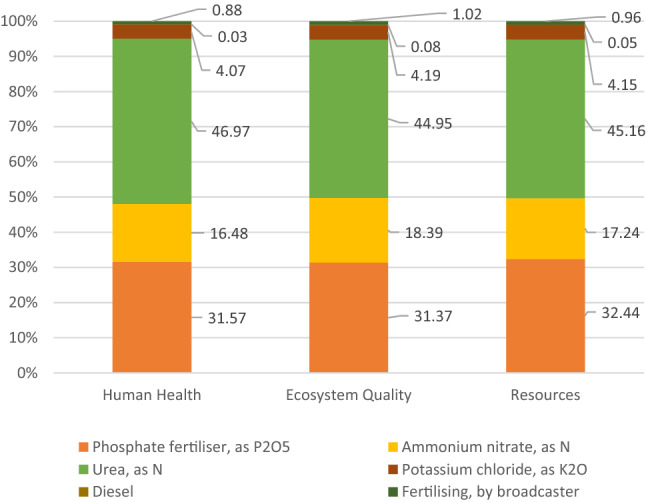
Structure of WF of the fertilization process in conventional carrot production as per individual impact categories.

Detailed WF results for the fertilization process in conventional carrot production are presented in Fig. 6. Among the individual factors shaping the environmental impact, what stands out is the consumption of urea, i.e. nitrogen (44.9–47.0% of the total impact in individual categories) and of phosphorus fertilizers, the impact of which is at 31.4–32.4%.

**Figure 7:**
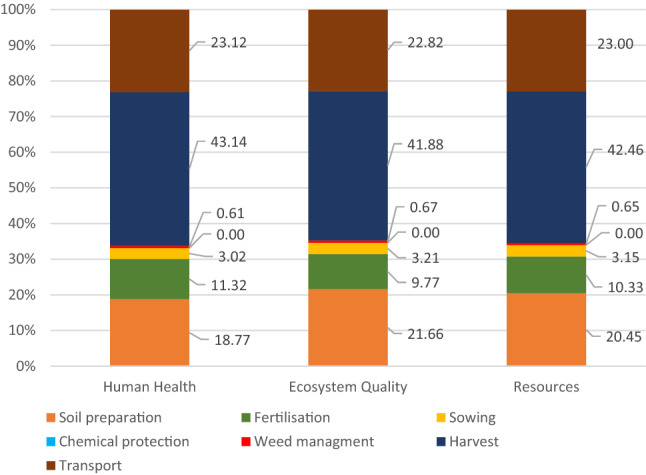
The structure of WF in individual impact categories in organic carrot production.

**Figure 8:**
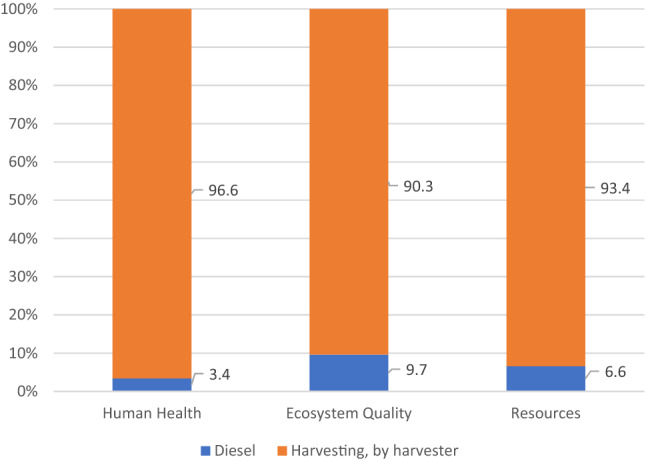
Structure of WF of the harvest process in organic carrot production as per individual impact categories.

In endpoint analysis, the impact of water use is generally related to specific endpoints in a given conservation area: Human Health, Ecosystems Quality or Resources^43^. The data in Table 3 shows WF in organic carrot production as per the three impact categories, and Fig. 7 shows its structure. The total WF values in each category are as follows: in the Human Health category—2.11E−06 DALY, in the Ecosystem Quality category—3.00E−08 species * year and in the Resources category—0.56 $ surplus. The above results are over five times lower compared to the footprint in conventional production (Table 2), and therefore it can be concluded that organic production not only enables the production of healthy carrot, but also has a very positive impact on the broadly understood environment. Upon analyzing the data from Tables 2 and 3, it can be observed that the environmental impact of fertilization treatment in organic production is over thirty times lower compared to the impact of fertilization in conventional production. Moreover, the fact that no pesticides are used means that the impact of chemical plant protection treatments is 0. Upon analyzing Fig. 7, it can be observed that the largest share in the total value of WF in individual impact categories is that of carrot harvest, from 41.9% (Ecosystem Quality) to 43.1% (Resources). The reason for such a significant environmental impact of carrot harvesting technology is the use of complex harvesters, as explained in Fig. 8. When calculating WF, both direct water consumption in a technology is taken into account, as well as indirect, related e.g., to the production of agricultural equipment used in the technology. The complexity of the machinery, the type of materials it is made of and the type of technology used in its production determine the WF.

**Table 3:** Environmental impact related to the use of water in organic carrot production per area unit (ha).

Specification	Total	Soil preparat.	Fertilisat.	Sowing	Chemicalprotection	Mechan. care	Harvest	Transport
Human health (DALY)	2.11E−06	3.96E−07	2.39E−07	6.39E−08	0.00E+00	1.30E−08	9.11E−07	4.88E−07
Ecosystem quality (species * year)	3.00E−08	6.49E−09	2.92E−09	9.61E−10	0.00E+00	2.00E−10	1.26E−08	6.84E−09
Resources ($ surplus)	5.61E−01	1.15E−01	5.80E−02	1.77E−02	0.00E+00	3.64E−03	2.38E−01	1.29E−01

The methodology for calculating WF is very diverse and includes many methods. Moreover, the results of research on WF related to the production of vegetable species presented in the literature often differ in terms of the analyzed system boundaries, production technology, irrigation, etc. Therefore, the possibility of a broad discussion of the results of WF of conventional and organic carrot production is limited.

The detailed structure of WF of the carrot harvesting process in organic farming is shown in Fig. 8. The use of machines, i.e. harvesters, has a decisive share (90.3–96.6%) in the total value of individual impact categories. The reason for this significant impact was explained above. Relatively high consumption of fuel during harvester operation contributes little to the water footprint structure. The use of diesel fuel has the highest impact on damage caused in the Ecosystem Quality category, with the lowest impact in the Human Health category.

## Conclusions

The LCA analysis showed that despite the lack of irrigation, carrot production requires significant water use and has a significant environmental impact, regardless of the technology used. The results of the research clearly show that the organic production of carrot brings benefits not only by supplying healthy vegetables to the market. It also benefits the environment, as evidenced by benign effect on ecosystems in various impact categories and good environmental indicators (WF, Human Health, Ecosystem Quality, Resources). For example, the WF of the production area is over five times lower in the case of organic farming (WF = 1.9 m^3^ ha^−1^), compared to conventional production (WF = 10.4 m^3^ ha^−1^). The value of WF in individual impact categories, i.e. Human Health, Ecosystem Quality and Resources, is the most significantly impacted by the fertilization process in conventional production (67.0–67.7%), and in organic farming, by carrot harvest (41.9–43.1%). The listed agricultural treatments with an unfavorable environmental impact in terms of water consumption can become the foundation for the environmental modernization of the production technologies. The significant differences in WF of fertilization processes in conventional and organic production reveal the great potential of organic fertilizers in terms of environmentally friendly vegetable production. Due to the significant impact of diesel consumption on WF during certain treatments, it seems advisable to modernize the production technology not only by replacing some treatments or production materials, but also by involving the use of less energy-consuming equipment. The results can be used to shape sustainable development plans in agricultural areas. It can also be used to outline policy directions regarding foreign trade in water-consuming agricultural products. Further research will focus on the development of various carrot production technology variants, adapted to local water resources, ensuring high yields on the one hand, and on the other, limiting the negative environmental effects of water use.
